# Access to Veterinary Drugs in Sub-Saharan Africa: Roadblocks and Current Solutions

**DOI:** 10.3389/fvets.2021.558973

**Published:** 2022-03-09

**Authors:** Glória Jaime, Alexandre Hobeika, Muriel Figuié

**Affiliations:** ^1^CIRAD, UMR MoISA, Montpellier, France; ^2^MoISA, Univ Montpellier, CIRAD, CIHEAM-IAMM, INRAE, Institut Agro, IRD, Montpellier, France; ^3^Eduardo Mondlane University, Maputo, Mozambique

**Keywords:** access to medicines (ATM), animal health, international pharmaceutical market, supply chain, Sub-Saharan Africa (SSA), veterinary drugs

## Abstract

**Background:**

Access to veterinary drugs for livestock has become a major issue over the last decade. Analysis has tended to focus on the demand for these products, while studies looking at the drivers behind their use generally focus on farmer behavior and interactions between veterinarians and farmers. However, the use of drugs also depends on structural factors that determine the functioning of the drug supply chain and farmers' access to the drugs. This article presents an overview of the factors that limit access to veterinary drugs in sub-Saharan Africa (SSA) as well as the international policy tools and arrangements that claim to improve it.

**Methods:**

We have conducted a scoping review of the scientific and grey literature as well as the publicly-available data from both the animal health industry and international organizations. We aimed to gather information on the veterinary drugs market in SSA as well as on the international norms, recommendations, guidelines, and initiatives that impact SSA farmers' access to these drugs.

**Findings:**

We highlight numerous barriers to veterinary drug access in SSA. The SSA market is highly dependent on imports, yet the region attracts little attention from the international companies capable of exporting to it. It suffers from a high level of fragmentation and weak distribution infrastructures and services, and is driven by the multiplication of private non-professional actors playing a growing role in the veterinary drug supply chain. The distribution system is increasingly dualized, with on the one hand the public sector (supported by development organizations) supplying small scale farmers in rural areas, but with limited and irregular means; and on the other side a private sector largely unregulated which supplies commercial and industrial farming systems. Different innovations have been developed at the international and regional levels to try to reduce barriers, such as homogenizing national legislations, donations, and vaccine banks. Alongside decades-old inter-state cooperation, many new forms of public-private partnerships and other hybrid forums continue to emerge, signaling the private sector's increasing influence in global governance.

**Conclusions:**

Policies on animal health would be bolstered by a better understanding of the drivers behind and the components of access to veterinary drugs in different regional and national contexts. Inequalities in drug access need to be addressed and a market-driven approach adopted in order to strengthen our understanding of what determines veterinary drug use at the farm level. Policies should balance the interests of the various stakeholders, being careful not to reinforce bias toward certain diseases deemed “interesting” and neglect others that could prove to be highly important for veterinary public health.

## Introduction

Over the last 30 years, integrative approaches have been adopted in health policies, placing interdependencies on a global scale and between species at the forefront with the Global Health and One Health paradigms. Global, cross-species interdependency has been pushed to the forefront of discussions through Global Health and One Health paradigms. This approach has been justified by the increasing prominence of emerging infectious disease risks, such as HIV/AIDS, Ebola virus disease, antimicrobial resistance (AMR), and Covid-19. Most of these risks emerged in developing countries due to increasing contact with reservoirs of pathogens in animals as well as flawed health systems.

Access to drugs is a core component of any health system or policy. In the last few years, multilateral organizations have pushed for the reinforcement of regulation of the trade and use of drugs within the context of the struggle against AMR. These efforts have highlighted the importance of tailoring policies to national contexts if they are to be effective (1). However, although access to drugs has been the subject of many academic works dedicated to human health, in particular within the context of developing countries (2–6), the animal health sector has received much less attention. Recent attention given to AMR in international and national policy has led to an increase in animal health studies (7–9).

The aim of this paper is to provide an initial general picture of the issues related to drug access in the context of livestock farming in SSA. We focus on SSA because it is the poorest region in the world and thus demonstrates the most salient issues regarding access to drugs, and most SSA countries bear a heavy burden when it comes to the economic and health impacts of animal diseases (10). Secondly, SSA is large enough to provide examples of a wide range of national situations.

When it comes to understanding the drivers of consumption of veterinary medicines, previous studies have tended to emphasize the role of demand—that is the “final” consumers, whether farmers or veterinarians. These studies are mainly published in veterinary journals not directly concerned with publishing social science research. The conditions under which drugs can be accessed and the importance of supply have been overlooked. Pioneering work on these issues in the social sciences has focused on Western countries (11–14). In SSA, a few studies have adopted a market-driven approach to veterinary drugs: this is partly the case for Bardosh et al. (15) in Uganda, Bessell et al. (16) in Tanzania, Kingsley (17) in Nigeria, and (18, 19) in Kenya. Available data on the veterinary drug supply chain have described the world market of veterinary drugs (20, 21), the regulation of this market (22–24), and the processes of harmonization of technical specifications at an international level (22, 25, 26), but they are to a certain extent outdated and only include brief references to SSA.

In studies on SSA specifically, more attention has been given to veterinary infrastructures, and their role in delivering services to low-income farmers in view of agricultural development (27–33). Some elements on access to drugs can be found indirectly in works focusing on specific animal health issues (e.g., bovine trypanosomiasis, tick-borne diseases, Newcastle disease, etc.) or products (vaccines, trypanocidal drugs). These studies describe the uses and misuses of drugs by farmers and animal health workers as a consequence of their knowledge and perceptions of diseases and drugs. They provide information on farmers' perceptions and self-assessment of veterinary drug-dispensing services [see for example Somda et al. (34) for Gambia; Enahoro et al. (33) for Ghana and Tanzania; Machila et al. (35, 37) and Higham et al. (36) for Kenya; Moffo et al. (8) for Cameroon; Soudre et al. (38) for Burkina Faso]. Many of these studies highlight farmers' lack of knowledge, awareness, or compliance (39, 40). Less common are studies that attest to the farmer's essential and positive role in animal disease management (41).

This paper is a scoping review based on the academic literature and publicly available grey literature. We aim to underline the main challenges sub-Saharan countries face in providing equitable access to veterinary drugs. We adopt here the definition of veterinary drugs proposed by the FAO (42), which includes: “drugs, insecticides, vaccines and biological products, used or presented as suitable for use, to prevent, treat, control or eradicate animal pests or diseases, or to be given to animals to establish a veterinary diagnosis, or to restore, correct or modify organic functions.” In this paper, we focus on drugs used for livestock (excluding pets), and on modern drugs (excluding traditional or ethnoveterinary medicines). According to the WHO (43), drug access is defined by the availability of drugs, including issues of quantity, regularity, quality and diversity, and affordability (or economic accessibility).

We present the information we gathered according to a socio-economic framework of supply chains. We consider access to drugs as the result of activities carried out by various entities (public and private) from the conception of a product to its final use, including the issue of residues. These activities include research and development, production, distribution, prescription, and use of drugs. The stakeholders and activities involved can be referred to as the veterinary drugs supply chain, using a broad understanding of the notion of a supply chain, which also includes all of the actors that contribute indirectly to the organization and functioning of the circulation of drugs, from molecules to residues, through the drafting of norms, rules, and recommendations.

This approach makes it possible for us to highlight the variations and inequalities between and inside countries, and the structural factors that limit the choices available to low-income farmers while minimizing the role of individual attitudes and perceptions as social determinants of consumption patterns. It looks at supply as a driver of consumption, and at policies on access to drugs, and regulation of their use.

After describing how the material used for this study was selected (Section Materials and Methods), we present the main socio-economic barriers to drug access in SSA (section The Many Factors That Limit Access to Veterinary Drugs in SSA) before presenting an overview of the contemporary political arrangements that have emerged at the international and regional level (Section International and Regional Arrangements for Improving Access to Veterinary Drugs) as part of efforts to improve access to veterinary drugs in SSA.

## Materials and Methods

Data were collected through a scoping review (44) of academic work and grey literature containing empirical material. The main objective was to map existing knowledge and to identify gaps in knowledge on veterinary drug access in SSA.

For the academic work, a scoping review was conducted using the scientific database Web of Science. The search terms used to identify publications were: [(drug^*^ OR medicin^*^ OR pharma^*^ OR access) AND (veterinary^*^ OR animal OR zoo^*^ OR husbandry OR livestock OR poultry OR sheep OR goat OR pork OR cattle) AND (trade OR use OR delivery OR service^*^) AND Africa AND (health OR disease OR epidemic OR epizoo^*^) NOT ethno]. These terms were searched in the topic (= title, abstract key word) and for the publication period 1975 to 2021, in all types of documents. From the 1,076 documents pre-selected, a first screening based on title and abstract and a second one based on the full texts, and 46 relevant documents were finally selected.

For the grey literature, we looked at the websites of different organizations involved in animal health in SSA and in some cases contacted them directly. This included the World Organization for Animal Health (OIE), Food and Agriculture Organization of the United Nations (FAO), AU-IBAR (the African Union – Interafrican Bureau for Animal Resources), ILRI (International Livestock Research Institute), World Trade Organization (WTO) as well as the Veterinary International Committee for Harmonization (VICH), GALVmed (The Global Alliance for Livestock Veterinary Medicines), HealthforAnimals (a non-profit, non-governmental organization representing companies and trade associations from developed and developing countries), pharmaceutical companies (Elanco Animal Health, Virbac, Zoetis, and MSD), and market research companies (Vetnosis, Mordor intelligence, Transparency market, Future Market insights). We collected data and technical reports describing the international veterinary drug market, veterinarian services, and drug distribution and use in SSA. Our material also includes recommendations, guidelines, norms, directives, and agreements related to veterinary drugs.

## Results and Discussion

We present and summarize here results concerning factors that limit access to veterinary drugs (understood in terms of quantity, diversity, adequacy, physical or geographic accessibility, affordability) by using a general framework of supply chains: overall market size, production, trade, and consumption. Subsequently, we present the contemporary repertoire of policy tools used to overcome these barriers.

### The Many Factors That Limit Access to Veterinary Drugs in SSA

What little information there is on the veterinary drugs market in SSA is difficult to access. Market information is not freely or wholly shared by the economic actors involved; information transmitted by national bodies to international organizations such as the OIE is not always publicly accessible, e.g., the OIE reports assessing the performance of the national veterinary services (PVS) or the veterinary legislation (VLSP). Moreover, any statistics that are publicly available are likely to only partly document the circulation of veterinary drugs, due to the market share held by informal products. “Illegal drugs” represent 20–30% of the market, according to HealthforAnimals (45). Additionally, the categories used to describe this market vary according to the source. These variations relate to the types of drug (insecticides, vaccines, biologicals, pharmaceuticals, feed additives), types of animals (pets, farmed animals), and geographic groups.

Nevertheless, these sources give an overall picture of the veterinary drug market in SSA: a small share of the world market, indicators of low use in some production systems, a limited local production of drugs (exemplified by the vaccines sector), weak distribution infrastructures and services, a lack of professionalization in the supply chains, and serious quality issues.

#### Production and Imports

The production of veterinary drugs is limited in SSA. Only a few countries have private drug manufacturers (mainly tertiary manufacturers), such as Bupo Animal Health (formerly Bedson) in South Africa and Cooper-K in Kenya, or the capacity to even partly supply neighboring countries. The production of veterinary drugs is often underpinned by public veterinary structures that focus on easy-to-produce generic medicines to support veterinary public health activities for small-scale farmers through vaccination campaigns or parasite control.

**Figure 1:**
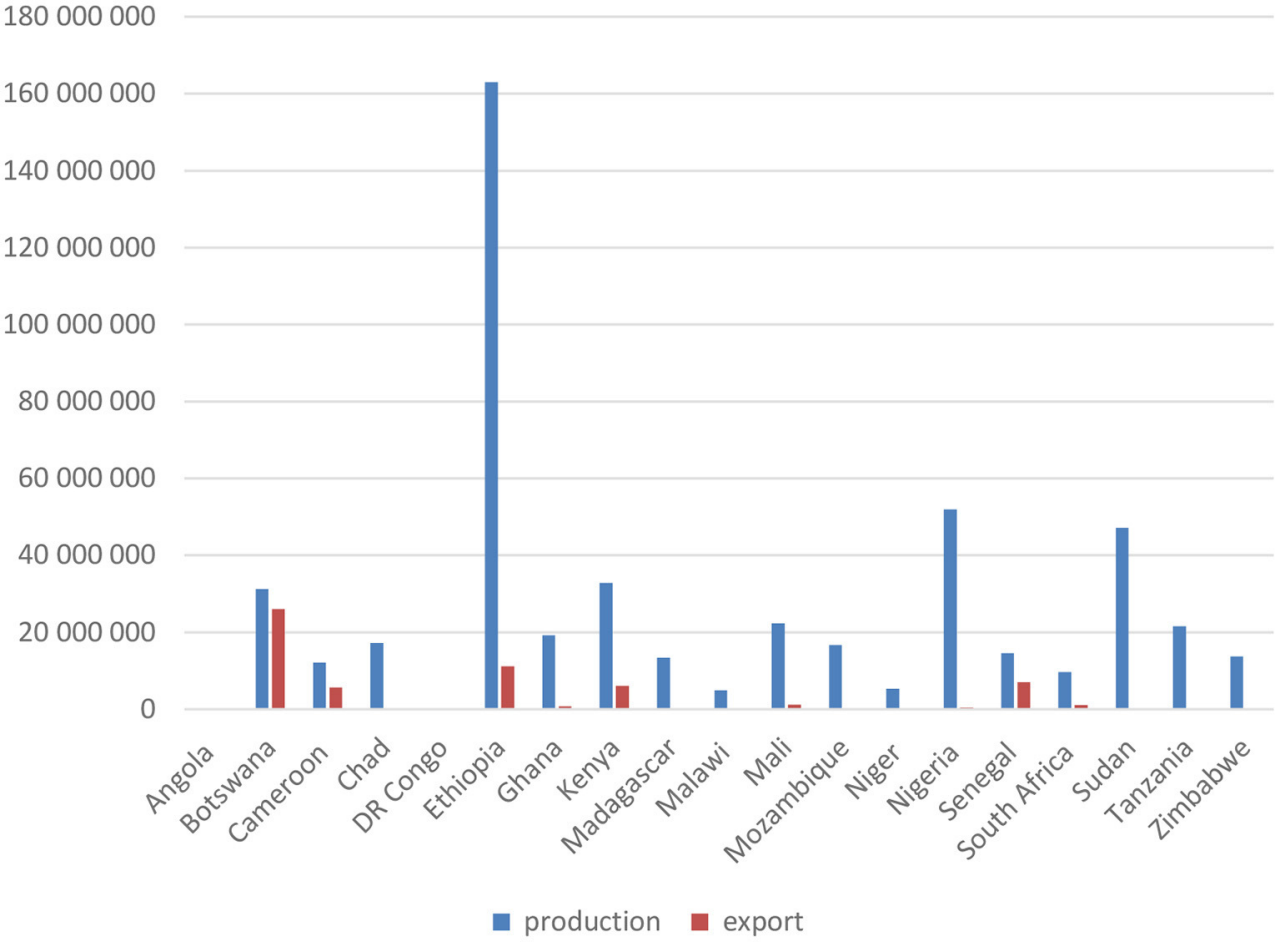
Production and export of vaccines for animals in sub-Saharan African countries (based on OIE Wahis database, average number of doses for 2014–2018).

Regarding the specific case of vaccines (see Figure 1), the information provided by the OIE Wahis database^1^ for 19 countries in SSA suggests 500 million doses a year were produced in the region during the 2014–2018 period, covering around 20 different types of vaccines. Ethiopia represents a third of this production, the vast majority of which was vaccines for poultry. The most widely produced vaccines were for Newcastle disease and anthrax (produced in 14 of the 19 countries where data is available), followed by the vaccine against *Peste des Petits Ruminants* (PPR) produced in 9 of the considered countries. Production mainly responds to national needs. Few countries have the capacity to export: the most salient exception is Botswana, a country that exports around 80% of its production and represents 43% of all recorded exports in SSA. Regional cooperation exists, such as the Pan-African Veterinary Center of the African Union (AU-PANVAC) in Ethiopia, which produces biological reagents for animal disease diagnosis and also provides independent quality control of veterinary vaccines.

The veterinary drugs market in SSA is dependent on imports from Europe, the US, Brazil, and, increasingly, China and India, with a complex organization between primary, secondary, and tertiary manufacturers and export and re-export processes that still need to be clarified by further research. Most of these products are imported by national distributors. Among the larger pharmaceutical companies, only a few have established branch offices in SSA, according to their annual reports and websites. Elanco Animal Health, Virbac, Zoetis, and MSD Animal Health have all established branch offices (subsidiaries) in South Africa. The Elanco Animal Health group, which acquired Boehringer Ingelheim's veterinary branch in 2016 and Bayer's in 2019, has the most extensive presence on the continent, with subsidiaries also in Angola, Kenya, Mozambique, Zambia, and Zimbabwe. The lack of harmonization in SSA national regulation, in particular when it comes to drug registration processes, contributes to a market fragmentation that discourages importation (46).

#### Weak Distribution Infrastructures and Services in the Public Sector

Infrastructure and services are necessary for the adequate distribution of drugs to their final users. Various papers underline how limited access to veterinary services is a major problem for livestock producers in sub-Saharan countries: see the issues of the OIE review dedicated to *Veterinary institutions in the developing world: current status and future needs* with a special focus on SSA countries in 2004 and the issue *Good governance and financing of efficient veterinary services* in 2012; as well as the recent review of Abakar et al. (32) on the status of veterinary services in the Sahel over the last 20 years. Other studies focus on a specific country or group of countries such as Kenya and Uganda (47–50), Tanzania (51), Central Africa (52), South Africa (53, 54), or Tanzania, Uganda, and Ethiopia (55).

In most countries in SSA, access to veterinary drugs was provided in the past by a centralized public sector inherited from the colonial period (31), managed by the veterinary profession and based on a populational approach to animal health (56). However, in the 1980s, under pressure from the World Bank, most developing countries adopted structural adjustment programs (SAPs) taking a market approach as the preferred means of providing services whilst at the same time reducing state expenditure. Impacts of the SAPs have been extensively analyzed and discussed in several international forums and publications in the decades following the reform, analyzing the consequences of the subsequent drastic shift of responsibility from the public to the private sector, including in the veterinary drug deliveries and veterinary services (30, 48, 52).

For example, Smith (52) indicates that the SAPs “imposed drastic reforms aimed at restructuring the public veterinary service and at privatization”; this process has been top-down and at times chaotic with no attempts made to find appropriate solutions for the diversity of production systems. Smith concludes that only a handful of countries and a small proportion of producers have benefited from this privatization. A successful example is that of cattle breeders in the Central African Republic, whose access to trypanocides (for the control of trypanosomiasis) was greatly improved. This success is attributed to the powerful cattle breeders' association determinant in driving the reform (52).

Veterinarians and para veterinarians^2^ are few and far between in SSA, see for example (57) in Ethiopia. Based on data from the OIE WAHIS database, we estimate that there are ~7.4 animal health professionals for every 100,000 inhabitants in SSA (made up of two veterinarians and 5.4 para-veterinarians). By comparison, there are on average 49 and 53 animal health professionals for every 100,000 inhabitants in the UK and US, respectively. Moreover, public services suffer from inadequate and unpredictable budgetary allocations and drug supply and have limited capacity to visit farmers. Their role as drug suppliers is restricted to the delivery of parasiticides and to vaccination, particularly during outbreaks. Rates of absenteeism are high and opportunities for career progress are limited. Some of these veterinarians work in parallel for private clinics, selling drugs and delivering therapeutic individual care for pets and farmed animals. This partly makes up for the absence of the private sector but also contributes to the blurring of lines between public and private services (27).

The performance of the veterinary authorities in regulating the circulation and use of veterinary drugs is also described as limited in many SSA countries, according to the PVS evaluation tool developed by the OIE. This tool includes a section on the technical authority and capability in relation to veterinary medicines and biologicals. Grading ranges from one (“The veterinary services cannot regulate veterinary medicines and biologicals”) to five (“The control systems for veterinary medicines and biologicals are regularly audited, tested and updated when necessary, including via an effective pharmacovigilance program”). In SSA, based on the currently available reports on the OIE website for 20 countries, two countries are graded level one (Guinea Bissau and Congo), 15 countries level two (Guinea, Ivory Coast, Benin, Mali, Mauritania, Namibia, Niger, RCA, Kenya, Seychelles, Rep of Sudan, South Africa, Togo, Chad, and Nigeria), two countries level three (Senegal and Swaziland), one country level four (Botswana), and none are graded level five.

This public sector weakness has a greater impact on low-income farmers, particularly in remote areas. It also limits the potential to face public health challenges requiring the intervention of public authorities, and regional or international coordination.

#### Challenges in the Development of Private Distribution of Veterinary Drugs

The number of public veterinary services has not been fully offset by the private sector, particularly with regards to the distribution of veterinary drugs in rural areas. These reductions have led to many failures in the supply of veterinary drugs and services (48, 49). Gehring et al. (58) indicate that in some villages in South Africa, the nearest accessible outlet for veterinary drugs was between 10 and 30 km. There is little incentive for private veterinarians and pharmacists to provide services in areas where the use of veterinary drugs per cattle head is low, purchasing power is limited, animals are widely dispersed, and transaction costs are high. Private veterinarians are more likely to commit to sectors where revenue is higher, such as the emerging market for pet health in cities or the burgeoning sector of intensive livestock farming in peri-urban areas (31, 52, 59, 60).

The privatization of veterinary services has contributed to the transformation of veterinary drugs and services from public goods to simple commodities. This privatization has an impact on drug availability: it favors the offer of drugs with high economic returns or those that respond to farmers' habits regardless of efficiency and adequacy. For example, Bardosh et al. (15) show that product availability in Uganda is dependent on what interests the animal health industry, which has led to higher sales in non-tsetse effective drugs.

Most studies on the provision of veterinary services conclude that there is a need for collaboration between the different stakeholders of veterinary services (public/private, donors) (32, 61), including farmers (41), as well as between human and animal health services (62, 63). More recently, to support veterinary drug delivery and services, new business models and institutional arrangements have emerged, such as cost recovery for public veterinary services, public/private partnerships, or contract farming. Experiences of contracted farming have been documented, for example, in the poultry and aquaculture sector in Burundi, Kenya, Rwanda, and Uganda (64).

#### The Multiplication of Non-professional Actors in Veterinary Drug Supply Chains

Gaps in delivery of veterinary services following the implementation of the SAPs have been partly filled by a variety of actors with basic knowledge or by other unqualified actors (65–69). Some studies, for example, that of Turkson (70) in Ghana, describe how shortages of practicing veterinarians see farmers taking the medication of their animals into their own hands.

Community animal health workers (CAHWs), sometimes referred to as the third sector (as opposed to the public or private sectors), have been trained to fill this gap, usually through the support of donors (49). They provide basic veterinary services to farmers in rural areas. Their formal knowledge consists of brief training from public veterinary services and NGOs (71). They are encouraged to develop a private veterinary drug supply system to finance their activities long term. Successful examples have been reported, for example in Kenya (18, 72). However, as public services, this CAHW-provided service also suffers from many constraints such as irregular supply, the low purchasing power of farmers, and transport difficulties (73, 74).

In various countries, the liberalization of veterinary drug distribution has also encouraged the emergence of alternative supply chains made up of a large number of middlemen (66, 69), mostly in peri-urban areas. In these areas, livestock farming is developing in conjunction with the increasing urban consumer demand for meat and a process of intensification supported by urban investors or by producer organizations (e.g., commercial poultry farmers' associations). The private markets for veterinary drugs have become concentrated in these areas. Private as well as public veterinarians (as part of a secondary activity) are involved in these private supply chains. Some of the individuals involved only have practical knowledge of drug use (e.g., commercial poultry farmers), while others do not have any knowledge at all but have capital they wish to invest in growing markets. Frequent failures observed in veterinary administration and regulation have left the private supply chains unregulated from imports to retail, and many drugs are sold without prescription. As a consequence, veterinary medicines can be found anywhere, anyhow (27). Gehring (75) describes a significant record of adverse reactions reported to the Veterinary pharmacovigilance center in South Africa due to inappropriate, extra-label uses of products by non-veterinarians.

#### Issues With Convenience and Quality of Available Drugs

The issue of veterinary drug accessibility also includes questions around convenience, suitability for local needs, and quality. As in the human health sector, (76), diseases endemic to Africa have received little attention from the pharmaceutical industry or research into disease epidemiology, which raises the issue of neglected animal diseases (77). The low level of training provided to CAHWs also limits both the convenience and diversity of available veterinary drugs. The role of CAHWs in delivering medicines is generally officially limited to drugs that represent the least potential for abuse, those with a broad-spectrum, and those that can be sold over the counter.

This lack of diversity, along with differences in price, may encourage the extra-label use of medicines, including use for other indications, methods of administration, species, age groups, and so on. This practice also includes the use of human medicines for animals, particularly when human medicines are more easily available and affordable, which can be the case when different countries adopt economic policies including low import taxes and grants aimed at improving access to human drugs. These uses give rise to inappropriate use of drugs, particularly in the absence of technical supervision and an effective regulatory framework. For example, veterinary services in Madagascar have reported injectable contraceptives intended for women (progestogens Confiance™, Pfizer), easily available at a low price, being used as an alternative for surgical castration of adult sows before culling (78). Misuse is also fostered by unsuitable packaging, for example, labels in foreign languages, or when small-scale farmers only have access to 1,000-dose packs Newcastle-disease vaccines, despite only having a relatively small number of animals.

Sub-standard and non-registered drugs are also an issue. The market for illegal drugs is estimated to be worth 400 million US dollars a year in SSA and North Africa and 1–2 billion US dollars worldwide (45, 79). Institutions for drug quality control are sorely lacking and only a few countries with significant production capacities (Botswana and Ethiopia) have properly equipped control laboratory facilities staffed by technically competent personnel, according to the aforementioned PVS tool. The lack of quality control and reliable certification of quality hinders farmers in their distinction between high- and low-quality drugs. Consequently, this deters sales of high-quality products, leading to an economic mechanism of adverse selection whereby bad products drive good products off the market (80).

Various issues with quality have been raised, from lower concentrations of active ingredients than that stated on labels to toxicity. According to a survey conducted in West Africa by the Interstate School of Veterinary Science and Medicine in Dakar and quoted by Le Minor (26), 67 and 69% of the veterinary drugs sampled in the formal and informal sectors, respectively, were of sub-standard quality. Of these sub-standard drugs, most were trypanocides and antibiotics (oxytetracycline). The sub-quality of trypanocides sold in SSA has been demonstrated by numerous studies. Bengaly et al. (81) provide an assessment of the quality of trypanocidal drugs sold in French-speaking countries in West Africa (Benin, Burkina Faso, Côte d'Ivoire, Mali, Niger, and Togo) in which “51.90% of the samples were non-compliant compared to the standards and were containing lower quantity [sic] of the active ingredient compared to the indications on the packaging.” Another study conducted by Tchamdja et al. (82) revealed a high proportion of trypanocides of sub-standard quality on the Togolese market (40%) and an even higher proportion (53.57%) for the sample collected from unofficial markets. The same problem is described by Tekle et al. (83) in Ethiopia, with 28% of trypanocidal drugs tested failing to comply with quality requirements. Vougat Ngom et al. (40) analyzed the quality of veterinary drugs sold in the Far North Region of Cameroon and concluded more positively that general quality was good, with concentrations often different but similar to that which is labeled and with no differences between vendors. Furthermore, they concluded that some differences in concentrations were likely the result of poor storage rather than intentional dilution and said the main problem in the region was poor compliance with recommended treatments among farmers.

#### At Farm Level: Low Availability and Affordability

Overall, the use of veterinary drugs in SSA is low. The global veterinary drug market has been described by Crosia (21) as globalized and dominated by less than ten American and European pharmaceutical firms. Recent analyses by market research firms have described the dynamic nature of the global veterinary pharmaceutical market thanks to a growing pet sector in Western countries and an increasing number of farmed animals in emerging Asian countries (84). SSA's contribution to this dynamic is difficult to calculate since data are scarce, but the omission of SSA in such market reports is also telling. Annual reports of the major companies and most market studies on veterinary pharmaceuticals do not refer to Africa or SSA or do so only indirectly through the category “rest of the word” (21, 85) or jointly within the category Middle East (84, 86). South Africa is the only country in SSA sometimes highlighted in market analysis (87). This indicates a lack of corporate interest in the SSA market. Other rare studies give an overview of the situation in specific countries in SSA: Messomo Ndjana (88) in his veterinary thesis on the distribution and quality of veterinary drugs in Cameroon; and Grasswitz et al. (20) in a report for UA-IBAR on the veterinary pharmaceutical industry in three sub-Saharan countries (Kenya, Uganda, and South Africa).

Depending on the source, the SSA market, along with other countries in North Africa and the Middle East, represents 1.7–7% of the global market (20, 21, 84). While there is a lack of recent data, past calculations have indicated that more than half of this market is concentrated in South Africa (20). These figures can be compared with the livestock population in SSA: according to FAO (10), SSA accounts for 14% of livestock worldwide (and North Africa and the Middle East represent 3.3%). Therefore, the average level of consumption of veterinary drugs per livestock unit in SSA can be estimated as between 12% (1.7/14) and 50% (7/14) of the world average use level.

Although the overall availability and diversity of veterinary drugs are low, significant differences exist between countries, farming systems, and animal species. A study conducted by GALVmed (unpublished, personal communication) based on a survey administered to local veterinarians in seven countries in SSA (Burkina Faso, Ghana, Kenya, Uganda, Nigeria, Senegal, Tanzania), documents the variations in farmer's access to drugs. Backyard poultry is the least “medicated” species in all countries studied. More than half of the backyard poultry farmers in Senegal, Uganda, Tanzania, and Kenya do not have any access to veterinary drugs. In the low-input small ruminant sector, veterinarians declared that half of the farmers did not have any access to drugs in Nigeria, and only had access to one type of drug (dewormers or antibiotics) in Burkina, Uganda, Tanzania, and Kenya. Access was assessed by veterinarians as very limited in Uganda and Tanzania, compared to Senegal, Burkina Faso, and Ghana. In Nigeria, the situation differs between species, with better access to treatments for small ruminants compared to those for poultry and cattle. The most accessible drugs were vaccines against Newcastle disease, antibiotics and anticoccidials for poultry, and antibiotics and vaccines against PPR for small ruminants (goats and sheep). In Tanzania, access is described as limited for the majority of farmers, however, a few cattle owners have access to a relatively large diversity of drugs (21 different drugs, which was the highest level of diversity reported by this survey for any species). Interestingly, in Uganda, antibiotics for backyard poultry are said to be more accessible than vaccines; and in the commercial sector, more than half of farmers only have access to antibiotics. Similarly, for small ruminants in Kenya, Uganda, and Tanzania, antibiotics were reported as being more accessible than vaccines.

Similarly, high variations in access and use between and within countries in SSA are shown in data focusing on antibiotics. The *Fifth annual report on antimicrobial agents intended for use in animals* edited by the OIE (89) indicates for 2017 an average consumption of 117.48 mg/kg of adjusted animal biomass for the 102 reporting countries, compared with an average of 30.35 mg/kg for the 24 reporting African countries (sub-Saharan and north-African countries). The specific case of Cameroon described by Mouiche et al. (90) shows large differences between species, from 213.32 mg/kg for poultry to 0.47 mg/kg for goats.

Finally, accessibility also depends on affordability. Most farmers in SSA have low purchasing power. According to the International Livestock Research Institute (91), poverty is widespread among livestock owners in SSA. Modern drugs are therefore less affordable for these farmers and the market opportunities are limited for supply chain stakeholders. Moreover, compared to emerging Asian countries engaged in what is commonly described as the “livestock revolution” (92), low-input farming systems remain predominant in SSA. For example, in 2011, SSA represented 2.1% of the world-intensive poultry production compared to 38% for China (and 46.8% for the whole East Asia and Pacific Region) (93). Low-input livestock production (including inputs such as veterinary drugs) is the main approach for farmers in pastoral areas who have limited and uncertain access to markets and cash and are exposed to external threats such as climate-related risks (94).

### International and Regional Arrangements for Improving Access to Veterinary Drugs

Different international- and regional-level institutional arrangements have emerged over time to help SSA countries improve drug access and coordinate and harmonize actions. This can directly improve access to veterinary drugs, promote the regulatory policies of international organizations, and mobilize pharmaceutical firms. Veterinary drug supply chains are framed by arrangements that have been promoted and institutionalized by international organizations such as the World Organization for Animal Health (OIE), the Food and Agriculture Organization (FAO) of the United Nations (UN), the Codex Alimentarius and the Veterinary International Conference on Harmonization (VICH), and the World Trade Organization (WTO) Sanitary and Phytosanitary Measures (SPS) Agreement (22–24). Standards set by the VICH and Codex Alimentarius also provide countries with a set of norms with which to regulate production, marketing authorizations, trade, and use of veterinary drugs (22, 24). Bilateral and regional agreements also contribute in the form of donations and vaccine banks. Aside from inter-state cooperation, we note a rapid increase in initiatives where the private sector plays a central role, in particular pharmaceutical companies. However, these arrangements rarely include Research and Development and are mainly focused on trade and veterinary advice rather than on the production side.

We focus here on the arrangements implemented at the international level, within a framework of international cooperation in which animal health is considered a global public good. Important efforts are also carried out at the national level, including, for instance, price subsidies, taxes, flexibilities in the Agreement on Trade-Related Aspects of Intellectual Property (TRIPS). However, they go beyond the scope of this paper.

#### Slow Harmonization of Regional and National Regulations

As mentioned above, many countries in SSA have very weak regulatory systems. For example, in Mozambique, there is no legislation addressing veterinary drugs, whilst in Angola, it appears that veterinary drugs are only superficially mentioned in legislation that mainly focuses on human medicines (95, 96). Moreover, regulations that are in place are not always effective, and the heterogeneity between countries restricts the opportunity for a regional market. To combat weaknesses in many national regulations—their lack of effectiveness and their heterogeneity inside the SSA—diverse initiatives have been implemented at the international or regional level. We present here the main international organizations participating in the regulation of drug access in SSA, as well as recently developed regional initiatives.

##### The Main International Institutions Regulating Veterinary Drugs

The OIE, established in 1924, is a major actor in this domain. It institutionalizes the sanitary norms for the international trade of animals and animal products, which member countries can use to prevent the introduction of diseases and pathogens without creating unjustified sanitary barriers (24, 97). For example, the Sanitary Code for Terrestrial Animals formalizes guidelines for the prudent and responsible use of antimicrobials. It also promotes the development of professional veterinary capacities and the involvement of veterinary services in the creation of regulations.

As with other commodities, the international trade of veterinary drugs is subject to norms set by the WTO. Through the SPS agreement, which came into force in 1995, the WTO seeks to reduce state use of non-tariff barriers that could be deemed unjustified and protectionist (98). The Codex Alimentarius, a joint program of the FAO and WHO established in 1963, focuses on food safety. It develops norms concerning the maximum residue limits of veterinary drugs in food, and by this means regulates the use of drugs in farming worldwide, throughout the supply chain (99, 100).

The VICH, the International Cooperation on Harmonization of Technical Requirements for Registration of Veterinary Medicinal Products, brings together regulatory authorities and the pharmaceutical industry in setting internationally recognized norms for veterinary drug registration and marketing authorizations (22–24). Established in the mid-1990s by industrialized countries (the EU, US, Japan), and inspired by the ICH (the International Council for Harmonization of Technical Requirements for Pharmaceuticals for Human Use), it is currently expanding its scope to become more global by including Nigeria, Uganda, Tanzania, and Zimbabwe. It aims to achieve greater international harmonization of registration requirements for veterinary drugs, to ease their circulation, and support their access (101).

##### The Regional Harmonization Initiatives From WAEMU, SADC and EAC

The African Union, through the AU-IBAR, is leading the harmonization of veterinary laws and regulations across various regional communities in Africa. This process is combined with harmonization in the domain of human health. GALVmed plays an important role in supporting this process. GALVmed is a non-profit NGO, with charity status, set up in the early 2000s by the UK's Department for International Development (DFID) and funded by the Gates Foundation. GALVmed takes inspiration from GAVI, the Vaccine Alliance, which works in the human health sector. It has been working since 2011 to promote drug access for small-scale livestock farmers in SSA (102). In particular, GALVmed is supporting the initiative “Harmonization of Registration Requirements for Veterinary Immunologicals and Development of a Mutual Recognition Procedure in East Africa Community (EAC)” which is funded by the Gates Foundation (46).

Other examples of ongoing initiatives aimed at homogenizing market authorization processes and quality control are given by the centralized system set up by the West African Economic and Monetary Union (WAEMU) in 2007 with the support of ANSES (the French Agency for Food, Environmental and Occupational Health & Safety) (103), and also by the adoption of the “Regional Guidelines for the Regulation of Veterinary Drugs in the Southern Africa” in 2011 (104). Despite these numerous initiatives, the harmonization process is said to be slow due to problems including weak national regulatory systems, financial problems, lack of institutional capacity, and challenges related to human resources (96).

#### Donations and Vaccine Banks

Donations and vaccine banks also contribute directly to the availability of veterinary drugs in SSA. In the human health area, Various authors (105–109) described the three main situations in which governments, companies, and NGOs donate drugs: emergency aid, development programs, de-stocking of unsold and almost expired drugs. Donations can also contribute to the improvement of drug access through a transfer of technology. For example, from 2018 to early 2021, the FAO and the EU donated equipment needed for the production of thermo-tolerant vaccines against PPR in Ethiopia. This donation has boosted the national production capacity and supported the National PPR Eradication Campaign (110, 111).

Donations can also be used to protect the commercial interests of the country making the donation. A donation of foot-and-mouth disease (FMD) vaccines made by the government of Botswana to Zimbabwe in 2017 is a case in point. The country donated over 473,200 doses of vaccines manufactured by the Botswana Vaccine Institute (BVI) in order to help Zimbabwe control outbreaks of FMD at their shared border (112).

The OIE (113) defines vaccine banks in its Manual of Diagnostic Tests and Vaccines for Terrestrial Animals (chapter 1.1.10) as “antigen or vaccine reserves, which can be of different types”. These banks enable the rapid supply of emergency stocks of vaccines in case of outbreaks, and lower delivery costs for systematic mass vaccination campaigns (114). According to Lombard and Füssel (115) and the OIE (116), banks are supplied by vaccine producers selected through international tenders. The cost of vaccines and their transportation to the recipient countries are generally borne by donors. To date, the OIE has set up two vaccine banks: one for avian influenza, and one for the PPR.

The avian influenza bank created in 2006, and now closed, received financial support from the EU through the PACE program and delivered 62,017 million doses of vaccines to six countries in SSA: Mauritania, Senegal, Egypt, Mauritius, Ghana, and Togo (117). In 2013, the OIE created the PPR vaccine bank under the Vaccine Standards and Pilot Approach to PPR Control in Africa Project (VSPA) with funding from the Gates Foundation and the World Bank through the Regional Sahel Pastoralism Support Project (PRAPS) (118). The Botswana Vaccine Institute (BVI) was chosen, after an international call for tender, to supply the PPR vaccines and the corresponding quantities of vaccine diluent (118). Different access modalities were deployed: direct purchase by a country (Togo), purchased through donors, or as part of regional programs (Burkina Faso, Ghana and Mali, Chad, Mauritania, Niger, and Burundi), or within the context of an emergency (Burundi in 2018). This vaccine bank has not only ensured the timely supply of high-quality vaccines complying with international standards, but it also facilitates the harmonization of PPR control methods in SSA. Regional organizations play a part as well. The Continental Veterinary Vaccine Bank was created in 2018 by the African Union and its Pan-African Veterinary Vaccine Center (PANVAC) with the support of the FAO, the OIE, the EU, the Gates Foundation, USAid, GALVmed, and certain countries (119). It mainly focuses on the prevention of a resurgence of Rinderpest.

#### Public-Private Partnerships

Over the last decade, public-private partnerships (PPPs) have become an increasingly common method of improving access to veterinary drugs. PPPs are defined as “a collaborative approach in which the public and private sector share resources, responsibilities and risks to achieve common objectives and mutual benefits in a sustainable manner” (120, 121). Recently, the OIE (121) published guidelines for PPPs in the veterinary domain. According to these guidelines, PPPs enable the development of animal health services, policies, and trade to a scale, quality, or degree of geographic coverage that would be unattainable for the public sector alone. PPPs can contribute to the improvement of access to drugs, reinforcement of veterinary services, encouragement of technology transfer agreements, and an increase in R&D into new drugs (121–123).

Over the last few years, different actors (governments, international organizations, NGOs, private companies, philanthropic foundations) have increasingly promoted the value of PPPs. At an international level, many authors in the humane health sector have documented the importance of PPPs and note their implementation as evidence of the increasingly proactive role played by the private sector in global decision-making processes, including in UN activities (108, 124–127). In the veterinary domain, their importance was further emphasized in the OIE Performance of Veterinary Services (PVS) pathway diagram (122), but a limited number of examples of PPPs are available.

The PPP initiated by the Gates Foundation and Zoetis in 2017 within the framework of the African Livestock Productivity and Health Advancement (ALPHA) initiative is one such example. The Gates Foundation pledged an investment of $14.4 million over 3 years (later extended to 5 years until 2022) to bolster the sustainable growth and development of the livestock sector in SSA (primarily in Nigeria, Ethiopia, Uganda, and, more recently, also Tanzania) (128). The partnership aims to improve access to veterinary drugs and services, provide training and education, and implement diagnostic infrastructure (128). Zoetis' role was to: establish basic infrastructure; increase the reliable supply of quality veterinary drugs, diagnostics, and services; develop veterinary laboratory networks and dialogue with government stakeholders to understand local requirements and needs, including regulatory issues (128). The governments of these countries were not directly involved in the partnership, but this example shows how the PPPs can be complementary to public action, which could provide some of the efficiency, management capacities, and culture of evaluation more commonly associated with the private sector.

PPPs can strengthen veterinary services in SSA. The PPP signed in 2011 between the Gates Foundation and Sidai Africa (a private company supplying livestock and crop inputs, and training to farmers and pastoralists across Kenya) pledged to build around 150 branded franchise outlets to facilitate the supply of good quality and affordable veterinary products to 300,000 livestock-keeping households in rural Kenya over a 4-year period (129). While this is not a direct partnership with a government, this PPP demonstrates how the Kenyan government has enabled the private sector to complement its provision of veterinary services and provide veterinary products to rural areas (120).

## Conclusion

The different sources mobilized in our paper show that despite differences between and within countries, the Sub-Saharan African drugs market as a whole holds little appeal for international pharmaceutical companies compared to other geographical areas such as emerging Asian countries. It remains peripheral in the global market for modern veterinary drugs, with the exception of South Africa where most of the market is concentrated. The market supply chains are largely unregulated and highly fragmented in terms of registration procedures and market authorization. The distribution chains are weak economically and lack professionals as a consequence of the wave of privatization of veterinary services seen in the 1980s. Therefore, in various countries, we see a dual system for veterinary medicines. On the one hand, the public sector, supported by development organizations, supplies small-scale farmers, mainly in rural areas, but with limited and irregular resources. It focuses on the distribution of vaccines and parasiticides through large-scale campaigns. On the other hand, the largely unregulated private sector supplies the growing market of commercial and industrial livestock farming It relies on private veterinarians, a variety of wholesalers, and retailers (pharmacies, agricultural stores, etc.) including unqualified ones, all tending to cluster in urban and peri-urban areas.

Arrangements have been implemented at the international level to improve drug access in SSA and the efficiency of drug supply chains. They provide “traditional” supports to the different functions of the national veterinary services. Significant efforts have also been made to support national legislation on veterinary drugs (in particular to include the issue of AMR), harmonization of the registration procedures of drugs in SSA, and different arrangements to improve availability (donation, vaccine banks) relying increasingly on PPPs and the involvement of pharmaceutical companies in the drafting and implementation of public policies.

Several conclusions can be drawn for AMR policies and on policies that intend to turn animal health services into a global public good. These policies need to be informed by a better understanding of the drivers behind and the components of access to veterinary drugs in different regional and national contexts. Analysis of what stimulates the use of veterinary drugs in animal farming should not rely too heavily on farmer-veterinarian interactions or on cognitive and psychological factors that shape individual behaviors. These factors are over-emphasized by the studies based on the KAP—Knowledge Attitude and Practices—methodology because the use of drugs by farmers depends greatly on their accessibility. First, there is a need to identify the reasons for low accessibility, which we can divide into low availability (geographic accessibility, potential drug deserts), quality (of drugs, advice, and medical equipment), and economic affordability. In particular, economic studies on affordability are essential if we are to understand the price formation process and how relative prices of drugs influence the decisions of stakeholders. Assessment of drug access should also include the capacity of the whole supply chain to face epidemics and emergencies. Secondly, evolutions in international policy arrangements for veterinary supply chains show the increasing role played by commercial actors in selecting which drugs are made available and under what conditions. This has been made possible by the weak regulation of supply chains and public veterinary services. Policies should balance the interests of the various stakeholders, being careful not to reinforce bias toward certain diseases deemed “interesting” while others, which may still be important for veterinary public health, are neglected.

## Data Availability Statement

The original contributions presented in the study are included in the article, further inquiries can be directed to the corresponding author/s.

## Author Contributions

GJ, AH, and MF: conception and design of the work, data collection, data analysis and interpretation, and drafting the manuscript. All authors contributed to the article and approved the submitted version.

## Funding

This research is part of the ROADMAP project (Rethinking of Antimicrobial Decision-Systems in the Management of Animal Production). It has received funding from the European Union's Horizon 2020 research and innovation program under Grant Agreement No. 817626.

## Conflict of Interest

The authors declare that the research was conducted in the absence of any commercial or financial relationships that could be construed as a potential conflict of interest.

## Publisher's Note

All claims expressed in this article are solely those of the authors and do not necessarily represent those of their affiliated organizations, or those of the publisher, the editors and the reviewers. Any product that may be evaluated in this article, or claim that may be made by its manufacturer, is not guaranteed or endorsed by the publisher.

## Footnotes

^1^OIE-WAHIS (OIE World Animal Health Information System) is a database providing worldwide data on the animal health situation and animal health capacities https://wahis.oie.int/.

^2^According to the OIE terrestrial code, “Para-veterinarians”, or “veterinary paraprofessionals” are professionals authorized by the veterinary status body, working under the direction and responsibility of veterinarians.
